# Clinical Value of ^99m^Tc-3PRGD2 SPECT/CT in Differentiated Thyroid Carcinoma with Negative ^131^I Whole-Body Scan and Elevated Thyroglobulin Level

**DOI:** 10.1038/s41598-017-19036-9

**Published:** 2018-01-11

**Authors:** Rui Gao, Guang-Jian Zhang, Yuan-Bo Wang, Yan Liu, Fan Wang, Xi Jia, Yi-Qian Liang, Ai-Min Yang

**Affiliations:** 1 Department of Nuclear Medicine, The First Affiliated Hospital of Xi’an Jiaotong University, Xi’an, 710061 China; 2 Department of Thoracic Surgery, The First Affiliated Hospital of Xi’an Jiaotong University, Xi’an, 710061 China; 3 Medical Isotopes Research Center, Peking University, Beijing, 710000 China

## Abstract

The aim of this study was to assess the usefulness of integrin imaging with ^99m^Tc-PEG_4_-E[PEG_4_-c(RGDfK)]_2_ (^99m^Tc-3PRGD2) single photon emission computed tomography (SPECT)/computed tomography (CT) in detecting recurrent disease in patients with differentiated thyroid cancer (DTC), negative radioiodine whole-body scan (WBS) and high serum thyroglobulin (Tg). Thirty-seven patients who underwent total thyroidectomy followed by radioactive iodine ablation and had negative radioiodine WBS but elevated Tg levels were included. ^99m^Tc-3PRGD2 SPECT/CT was performed 1 week after the negative diagnostic ^131^I WBS. Diagnostic performance indicators, including sensitivity, specificity, positive predictive value (PPV), and negative predictive value (NPV), for ^99m^Tc-3PRGD2 SPECT/CT was calculated. The correlations between SPECT/CT results and clinic-pathological characteristics were examined. In 30 (81.1%) of the 37 patients, ^99m^Tc-3PRGD2 SPECT/CT showed positive uptake. The sensitivity, specificity, PPV, and NPV of SPECT/CT to detect recurrent disease at follow-up were 96.6%, 75%, 93.3% and 85.7%, respectively. The sensitivity and PPV of SPECT/CT increased with increasing serum Tg levels. ^99m^Tc-3PRGD2 SPECT/CT showed high sensitivity and PPV in the detection of recurrence among DTC patients with higher Tg levels and negative WBS, and the probability of obtaining a positive SPECT/CT result was related with the level of Tg.

## Introduction

Differentiated thyroid cancer (DTC) is the most common endocrine cancer, representing 1% of all malignant tumors^1^. Total thyroidectomy followed by radioiodine therapy and further thyroid hormone replacement therapy is still the mainstay of DTC treatment^2^. The thyroid stimulating hormone (TSH) stimulated thyroglobulin (sTg) levels should be undetectable in DTC patients who have undergone total thyroidectomy and successful iodine-131 (^131^I) ablation^3–6^. Increased serum level of TSH-stimulated Tg, especially when higher than 10 ng/mL, is always an indicator of recurrent/metastatic disease^2,7^. Cervical ultrasonography, chest radiography, and whole-body scan (WBS) with ^131^I are recommended for the detection of possible disease recurrence^8,9^. However, ^131^I WBS has been reported to be negative in 20% of the patients with elevated sTg levels^10^. The patients with sTg elevation and negative iodine scintigraphy are defined as having TENIS syndrome (thyroglobulin elevation but negative iodine scintigraphy), and in such a condition, the introduction of some additional tools for imaging of recurrence is needed for further treatment planning, particularly locoregional interventions^11^.

Fluorine-18-fluorodeoxyglucose (^18^F-FDG) positron emission tomography/X-ray computed tomography (PET/CT) is an established imaging modality in cases with TENIS syndrome^12,13^. However, the probability of obtaining a positive FDG PET result in TENIS patients is unsatisfactory, especially in cases with the serum level of sTg lower than 30 ng/mL. In addition, the limited prognostic significance of a negative result also hampered its routine clinical use^14–18^. Therefore, alternative imaging modalities to ^18^F-FDG PET/CT are requested for the detection of metastatic disease in patients with TENIS syndrome^19^. Nowadays, integrin α_v_β_3_ has been considered as a valuable target for diagnosis and therapy of various malignant tumors^20^. Compared with ^18^F-FDG and other radiolabeled arginine-glycine-aspartic acid (RGD)-based peptides imaging, Technetium 99m-dimeric cyclic arginine-glycine-aspartic acid peptides with three polyethylene glycol spacers (^99m^Tc-3PRGD2) imaging holds the advantage of low cost, widespread availability, as well as favorable physical and imaging characteristics^21^. Previous studies have demonstrated its usefulness in tracing radio-refractory DTC lesions^22^, and it is of note that ^99m^Tc-3PRGD2 might be more valuable than ^18^F-FDG imaging in the diagnosis of lymph node metastasis^23^. Thus, we speculated that RGD imaging may hold a possibility to resolve the challenge in clinical decision making and surgical planning in patients with TENIS syndrome. To evaluate this hypothesis, here we assessed the usefulness of ^99m^Tc-3PRGD2 single photon emission computed tomography (SPECT)/computed tomography (CT) in patients who had negative ^131^I WBS and elevated serum sTg following total thyroidectomy and radioiodine ablation.

## Results

### Clinical Characteristics

The total number of patients included in this study was 37, with 9 being male and 28 being female. The clinical characteristics were summarized in Table 1. All patients received thyroidectomy and radioiodine ablation and the median cumulative radioiodine therapy dose was 5.8 GBq (range, 3.7–13.3 GBq). In all patients, the final diagnostic radioiodine scan was negative along with elevated sTg levels, and a negative neck US and chest radiography.

**Table 1:** Clinical characteristics of DTC patients with TENIS syndrome (n = 37).

**Characteristic**	**Value**
Age, median (range), y	42.5 (18–72)
Sex, F/M, n	28/9
Histopathological finding	
Papillary caner, n (%)	32 (86.5%)
Follicular cancer, n (%)	5 (13.5%)
Tumor size, median (range), cm	1.3 (0.1–4.7)
Multifocal tumor, n (%)	15 (40.5%)
Vascular/thyroid capsule invasion, n (%)	24 (64.9%)
Extra thyroidal spread, n (%)	22 (59.5%)
Central lymph nodes metastasis, n (%)	27 (73.0%)
Laboratory findings	
Tg with TSH stimulation, ng/mL	106 (13.5–480)
Tg with TSH suppression, ng/mL	17 (2.1–59.6)
Reference standard	
Histopathology, n (%)	10 (27.0%)
Positive post therapeutic ^131^I WBS, n (%)	9 (24.3%)
Follow-up with other images and/or Tg, n (%)	18 (48.6%)

DTC, differentiated thyroid cancer; TENIS, thyroglobulin elevation but negative iodine scintigraphy; y, years; Tg, thyroglobulin; TSH, thyroid stimulating hormone; WBS, whole body scan.

### Analysis of ^99m^Tc-3PRGD SPECT/CT Images

**Figure 1:**
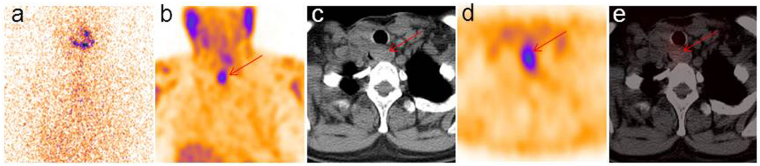
Typical SPECT/CT images of focal uptake patterns. ^99m^Tc-3PRGD2 imaging was performed 1 week after the negative diagnostic dose ^131^I WBS (**a**), to establish the presence of recurrence and/or metastatic disease in a TENIS syndrome patient. ^99m^Tc-3PRGD2 MIP SPECT image showed focal uptake in thoracic region (**b**), following SPECT/CT revealed increased radiopharmaceutical uptake corresponding to the enlarged lymph nodes (**c–e**, arrows).

**Figure 2:**
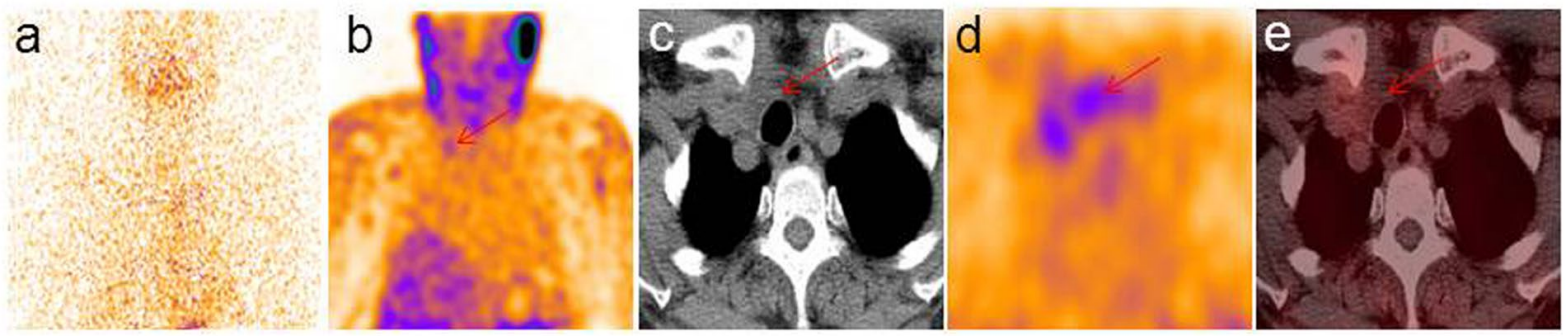
Typical SPECT/CT images of diffuse uptake patterns. The diagnostic dose ^131^I WBS showed negative result in the TENIS patient with sTg at 36.5 ng/mL (**a**). Diffuse uptake in the pretracheal space corresponding to a mass with unclear boundary was detected on ^99m^Tc-3PRGD2 SPECT/CT images (**b–e**, arrows). The sTg level of this patient was decreased after she received another ^131^I ablation, and her sTg level was 3.1 ng/mL at last follow-up.

In 30 (81.1%) of 37 patients, ^99m^Tc-3PRGD2 SPECT/CT showed positive uptake. Of 30 cases with positive uptakes on ^99m^Tc-3PRGD2 SPECT/CT, 22 patients presented with focally increased uptake, and the other 8 patients showed diffuse uptakes^24^. Focal uptakes referring to increased radiopharmaceutical uptakes corresponded to lesions with clear boundaries (Fig. 1). Diffuse uptakes refer to accumulations corresponded to masses with indistinct boundaries (Fig. 2).

Of 22 patients with focal uptakes, ^99m^Tc-3PRGD2 SPECT/CT images demonstrated increased radiopharmaceutical accumulations corresponding to the metastatic lymph nodes, lung nodules, and bone lesions in 17 patients. Nine of them were referred to lymphadenectomy; and the other 8 patients received empirical ^131^I therapy. Recurrent disease was subsequently revealed by pathological studies or post-therapy scans. The median sTg level of the 17 patients dropped from 125.0 ng/mL to 13.0 ng/mL at last follow-up. The remaining 5 patients showed focal uptake in thymus only. Surgical removal of the mediastinal mass in one patient provided histopathologic evidence of hyperplastic thymic tissue and led to decreased serum sTg level (62.2 ng/mL vs. 8.2 ng/mL at last follow-up). The other 4 patients with only mediastinal uptake were not assigned further treatment. No recurrence was detected during follow-up, but their stimulated Tg levels were consistently increased (median: 29.7 ng/mL vs. 15.5 ng/mL at last follow-up, n = 4).

Of 8 patients with diffuse uptakes, SPECT/CT images revealed ^99m^Tc-3PRGD2 accumulation in thyroid bed in 5 cases and in mediastinal mass with indistinct boundaries in 3 patients. Six of the 8 cases received another ^131^I ablation therapy and posttreatment iodine uptake was observed in 1 patient. Although the other 5 cases had negative post-therapy ^131^I WBS, they were considered true-positive as their sTg levels were decreased after treatment (median: 120.1 ng/mL vs. 9.6 ng/mL at last follow-up, n = 6). The other 2 cases with diffuse uptake did not receive treatment, because their sTg levels were gradually decreased without any treatment administered (45.0 ng/mL vs. 2.3 ng/mL and 19.7 vs. 1.8 ng/mL, respectively). It is of note that the T/B ratio of the non-focal uptake lesions was significantly lower than that of the focal uptake lesions (1.9 ± 0.9 vs. 5.8 ± 2.1, *P* < 0.0001).

Seven of 37 patients (18.9%) showed negative uptake on the ^99m^Tc-3PRGD2 SPECT/CT. Six patients whose sTg dropped spontaneously after SPECT/CT were considered true-negative. One patient who received ^131^I treatment and then had sTg dropped was considered false-negative. The sensitivity and specificity of ^99m^Tc-3PRGD2 SPECT/CT were calculated as 96.6% and 75%, and positive predictive value (PPV) and negative predictive value (NPV) were 93.3% and 85.7%, respectively.

At the end of this study, in the ^99m^Tc-3PRGD2-positive group (n = 30), 5 patients were alive with disease and the other 25 were alive with no evidence of disease. In the RGD-negative group (n = 7), all patients were alive and free of disease. The median entry sTg value of SPECT/CT-positive patients was significantly higher than that of the 7 SPECT/CT-negative patients (*P* < 0.0001). The sTg levels of ^99m^Tc-3PRGD2-negative group were still significantly lower than that of ^99m^Tc-3PRGD2-positive group after 6 months’ follow-up (*P* < 0.0001, Table 2).

**Table 2:** TSH and sTg levels in DTC patients who underwent ^99m^Tc-3PRGD2 SPECT/CT.

		RGD positive (n = 30)		RGD negative (n = 7)		*P* ^*^
		median	Range	median	Range	
Entry	TSH (uIU/mL)	88.7	47.1–100	87.8	62.4–100	0.53
	sTg (ng/mL)	114	15.5–480	19	13.5–62.4	<0.0001
6 mon Follow-up	TSH (uIU/mL)	91.5	68.5–100	90.8	84.7–100	0.54
	sTg (ng/mL)	19.5	0.2–86.8	3.1	0.2–9.7	<0.0001

^*^Comparison between RGD positive and negative patients. RGD, arginine-glycine-aspartic acid; TSH, thyroid stimulating hormone; sTg, TSH stimulated thyroglobulin; mon, month.

When stratified the 37 patients per their entry stimulated Tg levels (>30 ng/mL, or >10 but ≤30 ng/mL), we found that the sensitivity, PPV and NPV of ^99m^Tc-3PRGD2 SPECT/CT were higher in TENIS syndrome patients with sTg > 30 ng/mL (Table 3).

**Table 3:** Results of SPECT/CT per Tg levels under TSH stimulation.

	**sTg 10-30 ng/mL (n = 14)**	**sTg > 30 ng/mL (n = 23)**
TP	9	19
TN	3	3
FP	1	1
FN	1	0
Sensitivity (%)	90	100
Specificity (%)	75	75
PPV (%)	90	95
NPV (%)	75	100

sTg, TSH stimulated thyroglobulin; TP, true-positive; TN, true-negative; FP, false-positive; FN, false-negative; PPV, positive predictive value; NPV, negative predictive value.

### Correlation of SPECT/CT Results with Serum sTg Levels

**Figure 3:**
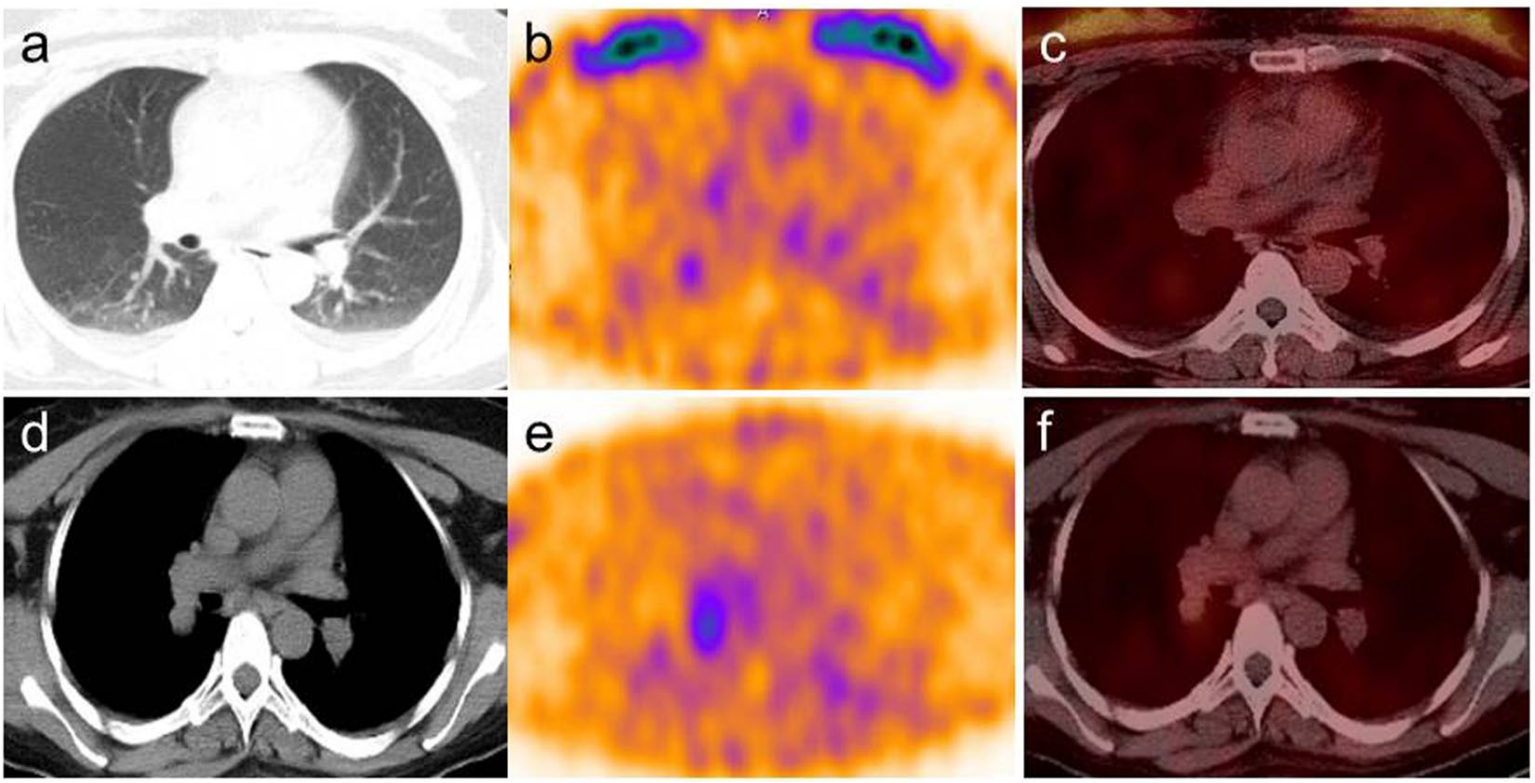
Representative ^99m^Tc-3PRGD2 SPECT/CT images of TENIS syndrome patients with different serum sTg levels. (**a**–**c**), Patient with stimulated Tg of 18.2 ng/mL demonstrated an uptake ratio of 1.5 in the right lung nodule; while (**d**–**f**), Patient with stimulated Tg of 58.5 ng/mL showed a T/B ratio of 4.8 in the right pulmonary nodule.

The median T/B ratio of lesions detected by ^99m^Tc-3PRGD2 SPECT/CT was 4.8 (range 1.4–13.2) in 30 patients with positive uptakes. We did not detect any lineal correlations between serum sTg levels and the T/B ratio in these patients (r = 0.280, *P* = 0.246). However, when we compare the T/B ratios of the patients per their clinical characteristics of the primary tumor, we found that ^99m^Tc-3PRGD2 uptake was significantly higher in patients with thyroid lesion diameter larger than 1 cm. As we expected, T/B ratio was significantly higher in patients with sTg > 30 ng/mL than in patients with sTg ranging between 10 and 30 ng/mL (Table 4, Fig. 3).

**Table 4:** Association of T/B ratios with clinical characteristics of ^99m^Tc-3PRGD2-positive DTC patients.

		^99m^Tc-3PRGD2 positive patients (n = 30)		***P***
		***N***	T/B ratio (median)	
Lymph node metastasis^*^	>3	26	5.1	0.071
	≤3	4	3.3	
Diameter of the lesion (cm)^†^	>1	26	5.3	0.036
	≤1	4	2.4	
Thyroglobulin (ng/mL)^‡^	>30	20	8.9	0.006
	10–30	10	2.6	
TSH (uIU/mL)^¶^	>80	27	5.3	0.167
	40–80	3	3.8	

^*^Number of lymph nodes with metastatic disease detected in primary surgery; ^†^diameter of the primary tumor; ^‡^serum Tg level under TSH stimulation at the time of SPECT/CT scan; ^¶^TSH level at the time of SPECT/CT scan. T/B ratio: tumor-to-background ratio; TSH, thyroid stimulating hormone.

## Discussion

Whole-body ^131^I scintigraphy and serial sTg measurements are standard methods for follow-up in patients with DTC^8,9^. However, a negative ^131^I scintigraphy result in a sTg-positive patient is a diagnostic and therapeutic challenge^11^. Using PET/CT in this group of patients is widely accepted, however, low sensitivity observed in patients with slightly increased sTg levels reflects that higher Tg level may be required to achieve a better FDG-PET accuracy^14–17,25,26^. Recent studies have suggested ^99m^Tc-3PRGD2 as a promising modality for guiding diagnosis and treatment of metastatic DTC lesions^22,27^. As far as we know, the present study is the first application of RGD imaging in detecting the possible recurrence or metastatic lesions in DTC patients with TENIS syndrome.

There are no definite guidelines regarding detection of recurrence in imaging studies. A consensus was reached that, as performed in this study, confirmation of the image results should be taken with histological examinations, other imaging findings, and the evolution of the Tg and anti-Tg levels in follow-up^13–17,22,25,26^. However, a diversity of stimulated/suppressed Tg levels were adopted as cut-off values in daily practice. Some researchers proposed non-recurrence as sTg levels spontaneously dropped below 2 ng/mL at follow-up^15,17^, while others suggested that patients with decreased sTg levels lower than 10 ng/mL could be taken as true negative^25,26^. In this study, patients with sTg levels consistently elevated, or decreased lower than 10 ng/mL, and/or non-stimulated Tg falling to less than 2 ng/mL for more than 6-month post treatment were grouped as true positive. According to this criterion, the result demonstrated that 81.1% of 37 TENIS syndrome patients showed positive uptake on ^99m^Tc-3PRGD2 SPECT/CT imaging, and 28 cases were true-positive. The sensitivity and PPV reached 96.6% and 93.3%, respectively, indicating that ^99m^Tc-3PRGD2 SPECT/CT is a sensitive imaging modality in tracing metastatic lesions in DTC patients with TENIS syndrome.

Integrin α_v_β_3_ is believed to play an essential role in tumor invasion and metastasis. Thus, a focal lesion found in ^99m^Tc-3PRGD2 SPECT/CT was supposed to have a more aggressive course and an appropriate therapy is needed. In our study, 22 of 30 SPECT/CT-positive cases demonstrated focal uptakes, and possible metastatic disease in lymph nodes, lung nodules, and bone lesions were revealed in 17 patients. The sTg level was decreased after treatment and 12 out of the 17 patients were alive with no evidence of disease at the end of this study. Interestingly, among the 22 cases with focal uptakes, 11 cases demonstrated ^99m^Tc-3PRGD2 accumulations in enlarged thymus. The findings regarding the thymus uptake, which would be reported in a later manuscript, proposed that the angiogenetic activity in hyperplastic thymus was related with the consistently elevated serum Tg levels in TENIS syndrome patients. Thus, the uptake in the thymus was also considered true positive in the 5 patients with mediastinal uptake only. Further intervention was suggested for patients with RGD uptake outside thymus, while close follow-up for patients with only mediastinal uptake.

Eight of 26 patients with positive uptake on ^99m^Tc-3PRGD2 SPECT/CT showed a diffuse uptake pattern in this study^24^. Although T/B ratio of these patients with diffuse uptake was significantly lower than that of patients with focal uptakes, metastatic disease was still suggested in 6 of them in the follow-up. Integrin α_v_β_3_ is involved in angiogenesis and highly expressed on the surface of activated endothelial cells in newly formed blood vessels^28–30^. It has been reported that integrin α_v_β_3_has pro-metastatic properties and integrin α_v_β_3_ imaging is considered to have the advantages as helping predicting the metastatic characteristic of tumor^23,31^. Therefore, it is reasonable to hypothesize that the diffuse uptakes, which showed lower integrin α_v_β_3_ expression level as compared to the focal uptakes, might indicate pre-clinical metastatic of the tumor^32,33^. The upregulated integrin α_v_β_3_ expression may trigger the tumor cell migration and neovasculature formation, whereas its amount is too small to form a mass with clear boundaries on the conventional radiological tests or concentrate enough RGD as focal uptakes^22,23^. Further clinical and experimental studies are needed to validate this possibility.

Although we did not detect any lineal correlations between serum sTg levels and the T/B ratio in TENIS syndrome patients, we found that ^99m^Tc-3PRGD2 imaging performed better in detecting recurrent/metastatic diseases in DTC patients with higher sTg levels (sensitivity reached 100% in cases with sTg >30 ng/mL *vs*. 90% in patients with 10–30 ng/mL sTg), i.e. it detected all the recurrences/metastasis in patients with sTg higher than 30 ng/mL. It is noticeable that in 14 DTC cases with sTg between 10 and 30 ng/mL, ^99m^Tc-3PRGD2 SPECT/CT detected recurrent/metastatic diseases in 9 patients. The sensitivity and specificity also reached 90% and 75%, respectively, which suggested ^99m^Tc-3PRGD2 SPECT/CT as a sensitive imaging modality in tracing metastatic lesions in TENIS patients. These data guaranteed further application of RGD imaging in TENIS patients, even in cases with mildly increased sTg levels. It has been indicated in a previous study that tumor size is correlated with integrin α_v_β_3_ accumulation on the imaging^22^. Our data also showed that the T/B ration was significantly higher in patients with a primary tumor size > 1 cm than in patients with tumor size ≤ 1 cm. In accordance with our results, some studies have reported tumor size as an independent predictor for poorer disease-specific survival^15,34,35^. We did not found extra thyroidal extension as an indicator for ^99m^Tc-3PRGD2 uptake in these patients, indicating that more complicated mechanisms might be involved in DTC metastasis.

A limitation of our study was the sample size was relatively small and the follow-up period is quite limited. Further studies with larger groups of patients are required to validate our findings. Half of the patients were confirmed by follow-up and the lack of pathological confirmation in these patients was another limitation of this study.

### Conclusions

^99m^Tc-3PRGD2 SPECT/CT showed high sensitivity and PPV for the detection of recurrence in DTC patients with negative radioiodine WBS but elevated sTg levels. In addition, our results suggested that the probability of obtaining a positive SPECT/CT result was positively correlated with the serum level of sTg.

## Materials and Methods

### Patients

A total of 37 DTC patients (9 males and 28 females; median age at the time of diagnosis, 42.5 years; range, 18–72 years) with negative ^131^I WBS despite elevated Tg levels on TSH stimulation were enrolled in this prospective study and underwent ^99m^Tc-3PRGD2 SPECT/CT imaging to establish the presence of thyroid bed recurrence or regional/distant metastatic disease in our institute. All included patients fulfilled the following criteria: (1) absence of previously detected metastatic disease in follow-up, i.e. negative cervical ultrasonography and chest radiography, (2) TSH-stimulated Tg level >10 ng/mL, (3) negative diagnostic ^131^I WBS and (4) absence of increased anti-Tg antibody levels^15^. Because the patients may receive further treatments if the Tg concentration kept increasing, the cases with serious comorbidities that may influence the uptake and/or excretion of ^131^I were excluded. This study was approved by the Ethics Committee of the First Affiliated Hospital of Xi’an Jiaotong University, and all participating patients provided an informed written consent. All methods were performed in accordance with the relevant guidelines and regulations.

### Whole-body scans with ^131^I

The standard procedure for ^131^I WBS is described as follows^3^. A patient was advised to be withdrawn from thyroxine supplementation 1 month before the scan and follow a strict iodine-restricted diet during this period to achieve TSH stimulation, which was above 40 IU/mL in all cases. Scans were performed with a large field gamma camera in whole-body scanning mode using a high-energy collimator. Anterior and posterior whole body scans were obtained 48 h after oral administration of 185 MBq ^131^I.

### Serum determination of thyroglobulin

Serum TSH, sTg, and anti-Tg antibody levels were measured by radioimmunoassay (GASK-PR, CIS-Bio International, subsidiary of Schering S.A., Gif-sur- Yvette, France).

### ^99m^Tc-3PRGD2 SPECT/CT

^99m^Tc-3PRGD2 SPECT/CT imaging was performed 1 week after the negative diagnostic dose ^131^I WBS. L-Thyroxine replacement was withdrawn 4 weeks before the ^131^I WBS.

Synthesis of the labeling precursor, kit preparation and subsequent ^99m^Tc-labeling were performed as previously described^36^. ^99m^Tc-3PRGD2 SPECT/CT and planar imaging were performed 5–54 months (median 15.31 months) after the last radioiodine ablation. The scanners were dual-head gamma cameras, using low-energy high-resolution collimators and a 20% energy window centered at 140 keV. SPECT and coregistered CT were performed 0.5 h after the intravenous injection of 11.1 MBq/kg (0.3 mCi/kg) of ^99m^Tc-3PRGD2. SPECT/CT (matrix 128 × 128 pixels, zoom 1.0, 30 s/frame/6°) of the neck and chest was performed with patient in the arm up position, followed by CT scan (120 kV, 160 mAs) with the same scan range of SPECT tomography. The Dicom image files of each patient were saved in optic discs and transferred to a Xeleris 2.0 workstation (GE healthcare) for centralized reconstruction, reading and analysis.

^99m^Tc-3PRGD2 images were visually interpreted by consensus of 3 experienced nuclear medicine physicians regarding SPECT/CT fusion and CT images. A positive result was defined as the accumulation of ^99m^Tc-3PRGD2 above its normal level in the surrounding tissue, excluding physiologically increased uptake. For semi-quantitative analysis, tumor-to-background (T/B) ratios of SPECT images were measured and calculated by the same person using a consistent standard. When more than one lesion was detected in one case, the highest T/B ratio was adopted for further analysis^23^.

### Follow-Up

The patients were followed up for 6–30 months (median 17.5 months) after the ^99m^Tc-3PRGD2 SPECT/CT imaging was performed. The SPECT/CT findings were compared with histopathological findings, serial radiological or clinical follow-up^23,36^. Patients were regarded as recurrence positive if either (1) histopathological examination by either biopsy or reoperation showed recurrence, (2) posttreatment WBS after empirical ^131^I therapy was compatible with recurrence or distant metastasis, (3) sTg levels showed consistently elevated levels at follow-up, whether ^131^I treatment was performed or not, (4) other imaging modalities displayed recurrent disease at follow-up, or (5) sTg levels dropped following empirical ^131^I therapy (non-stimulated Tg < 2 ng/mL, or TSH-stimulated Tg values < 10 ng/mL for more than 6 months). Patients were regarded as recurrence negative if either, (1) histopathological examination by either biopsy or reoperation excluded recurrence, or (2) other imaging modalities did not display recurrent disease and sTg levels spontaneously dropped. Using this definition of recurrence, we evaluated the diagnostic value of ^99m^Tc-3PRGD2 SPECT/CT to disclose recurrent disease in our patients^2,10–14,37^.

The SPECT/CT results were correlated with Tg levels and clinical and histopathologic characteristics of the primary tumor. In addition, we also stratified these patients per their entry stimulated Tg levels (>30 ng/mL, or >10 but ≤30 ng/mL) and evaluated the correlation between sTg levels and the ^99m^Tc-3PRGD2 SPECT/CT results^14^.

### Statistical analysis

Data analysis was performed using SPSS for Windows, version 12.0 (SPSS, Inc., Chicago, IL, USA). The difference of changes in tumor to T/B ratios on SPECT/CT between different groups was analyzed using the Student *t*-test. Categorical data were analyzed by Pearson’s Chi-square test. Degrees of association between continuous variables were calculated by Spearman’s rank correlation analysis. A *P* value < 0.05 was considered statistically significant.

### Data availability

The datasets generated during and/or analyzed during the current study are available from the corresponding author on reasonable request.
